# The dangers of mass drug administration of albendazole in Nepal, a Neurocysticercosis-endemic region

**DOI:** 10.1186/s40794-020-00122-2

**Published:** 2020-10-23

**Authors:** Gaurav Nepal, Ghanshyam Kharel, Yow Ka Shing, Rajeev Ojha, Sujan Jamarkattel, Jayant Kumar Yadav, Himani Vyas, Dhiraj Poudyal, Ranjit Sah

**Affiliations:** 1 Maharajgunj Medical Campus, Tribhuvan University Institute of Medicine, Maharajgunj Rd, Kathmandu, 44600 Nepal; 2 Department of Neurology, National Academy of Medical Sciences, Mahaboudha, Kathmandu, 44600 Nepal; 3 Department of Internal Medicine, National University Hospital, 5 Lower Kent Ridge Rd, Singapore, 119074 Singapore; 4 Department of Neurology, Maharajgunj Medical Campus, Tribhuvan University Institute of Medicine, Maharajgunj Rd, Kathmandu, 44600 Nepal; 5 Department of Infectious Diseases, Tufts Medical Centre, 800 Washington St, Boston, MA 02111 USA; 6 All India Institute of Medical Sciences, Sri Aurobindo Marg, Ansari Nagar, New Delhi, Delhi, 110029 India; 7 Department of Microbiology, Maharajgunj Medical Campus, Tribhuvan University Institute of Medicine, Maharajgunj Rd, Kathmandu, 44600 Nepal

**Keywords:** Neurocysticercosis, Albendazole, Soil-transmitted helminths, Lymphatic filariasis

## Abstract

The majority of cases of Neurocysticercosis (NCC) are asymptomatic. Injudicious use of antihelmintics like albendazole (ALB) can cause cyst degeneration and perilesional inflammation, thus rendering asymptomatic individuals symptomatic with seizures, headache, vascular events, or cerebral edema. Mass drug administration (MDA) using ALB is a very common practice in developing countries like Nepal to contain transmission of soil-transmitted helminths (STH) and lymphatic filariasis (LF). Although the benefits of ALB-based MDA in the general population cannot be undermined, there can be severe consequences in certain groups, especially those with latent NCC. In this commentary, we discuss the effect it may have on such patients, and suggest potential solutions.

## Commentary

Deworming campaigns target soil-transmitted helminths (STH) including the roundworm *Ascaris lumbricoides*, the hookworm *Ancylostoma duodenale*, and the whipworm *Trichuris trichirua*. In Nepal, the prevalence of STH infections ranges from 3.3 to 51.5%, and they manifest with morbidities such as delayed physical and mental development, anemia, and protein-energy malnutrition, which can significantly affect developing children [1]. Lymphatic filariasis (LF), another major public health concern, is a vector-borne disease mainly caused by *Wuchereria bancrofti* and transmitted by Culex mosquitoes. Nepal sees an average LF prevalence of 13%, with associated complications such as filarial hydrocele, scrotal elephantiasis, and lymphorenal fistula, all of which contribute to disability and social isolation [2].

It naturally followed that Nepal adopted a nationwide “Vitamin A Plus” capsule distribution campaign to target STH, designed with the administration of vitamin A alongside a single dose of albendazole (ALB) every 6 months to deworm children aged 6 to 59 months. The program started in the 1990s and eventually expanded to the entire nation in 2004 [1]. To address LF, a mass drug administration (MDA) regimen comprising diethylcarbamazine (DEC) and a single dose of ALB was implemented in all of Nepal’s endemic districts. The LF MDA program started in the Parsa district in 2003, reached a national scale in 2013, and has since seen the administration of over 100 million doses of DEC and ALB [2].

Despite the success of the aforementioned programs, they were never designed to target cysticercosis, a parasitic infection with a predilection for the nervous system caused by ingestion of *Taenia solium* eggs shed in the faeces of a human tapeworm carrier. Cysticercosis is endemic in Nepal, which holds a taeniasis prevalence of 43% in its central region and 10–50% across the different ethnic groups including the Magars, Sarkies, Darai, and Bhote [3]. Injudicious use of antihelmintics can be potentially detrimental in such places [4]. A single dose of ALB can render asymptomatic cysticercosis carriers symptomatic via latent cyst degeneration and perilesional inflammation. This can manifest as epilepsy, headache, vision loss, encephalopathy, or vascular events [5]. In a case series review examining five asymptomatic NCC patients who began experiencing neurologic symptoms after ALB use, four of them suffered seizures and one died from meningoencephalitis [6]. Although the exact prevalence of neurologic manifestations in such asymptomatic carriers is not known, adverse effects have been reported in the literature [5, 6].

We offer two possible solutions to address this issue. Firstly, point-of-care diagnostic tools that detect parasite-related antigen or antibody in serum can be used to diagnose NCC and/or taeniasis in all individuals participating in MDA [7]. If the serology is positive, the patient should be referred to a tertiary care centre for appropriate evaluation and management. As in the usual treatment of NCC, ALB will have to be administered concomitantly with high-dose steroids to control paradoxical aggravations of inflammation with standby anti-epileptics in the event of seizures [5]. If the test is negative, however, standard MDA should be administered. Considering the current primary healthcare set-up in Nepal and the possibility of leveraging on facilities and centers already used for MDA, the use of such diagnostic tools is feasible and assimilable. Despite this, practical barriers including the cost of screening, social attitudes towards screening, and the distribution of healthcare centers across the country may affect the seamless incorporation of diagnostic tools in such campaigns.

Secondly, alternative drug regimens excluding the use of ALB can be employed. For the roundworm, hookworm, and whipworm, the non-azole Tribendimidine (TRB) has similar egg-reduction rates (ERR) compared to ALB and has demonstrated an excellent safety profile. Deworming campaigns utilizing TRB can potentially treat STH as effectively without the side effect of treating cysticercosis [8]. For LF, a DEC-only regimen is a good alternative to the current collateral use with ALB as effected in Nepal. The scientific efficacy of DEC-fortified salt is well-established and pilot studies from Brazil, Haiti, India, and Tanzania have all shown that DEC-fortified salt reduces microfilaremia [9]. China, which once had the largest number of LF patients, safely and successfully employed DEC-fortified table salt and DEC-only MDA in achieving effective elimination of LF, proving its efficacy as a single agent [10]. One potential downside is the fact that a single-drug regimen would require an increased number of rounds of MDA to achieve eradication, as evidenced in modelling studies [11]. Additionally, factors such as cost, safety profile, and local perspectives toward the alternative need to be explored and analyzed prior to the implementation of such schemes on a national scale.

## Conclusion

Consideration of alternative regimens with appropriate use of point of care diagnostic tests can potentially reduce the side effects of ALB-based MDA in cysticercosis-endemic regions such as Nepal. We believe MDA should be a means of delivering safe and efficacious treatment. More research is warranted to define the scale and severity of the above problem and to guide the re-designing of current MDA regimens in Nepal.

## Data Availability

Not applicable.
